# Gold Nano-Island Platforms for Localized Surface Plasmon Resonance Sensing: A Short Review

**DOI:** 10.3390/molecules25204661

**Published:** 2020-10-13

**Authors:** Simona Badilescu, Duraichelvan Raju, Srinivas Bathini, Muthukumaran Packirisamy

**Affiliations:** Micro-Nano-Bio Integration Center, Optical-Bio Microsystems Laboratory, Department of Mechanical and Industrial Engineering, Concordia University, Montreal, QC H3G 1M8, Canada; duraich30@gmail.com (D.R.); bathini.srinivas3@gmail.com (S.B.); mpackir@encs.concordia.ca (M.P.)

**Keywords:** dewetting, nano-islands, gold, sensing

## Abstract

Nano-islands are entities (droplets or other shapes) that are formed by spontaneous dewetting (agglomeration, in the early literature) of thin and very thin metallic (especially gold) films on a substrate, done by post-deposition heating or by using other sources of energy. In addition to thermally generated nano-islands, more recently, nanoparticle films have also been dewetted, in order to form nano-islands. The localized surface plasmon resonance (LSPR) band of gold nano-islands was found to be sensitive to changes in the surrounding environment, making it a suitable platform for sensing and biosensing applications. In this review, we revisit the development of the concept of nano-island(s), the thermodynamics of dewetting of thin metal films, and the effect of the substrate on the morphology and optical properties of nano-islands. A special emphasis is made on nanoparticle films and their applications to biosensing, with ample examples from the authors’ work.

## 1. Introduction

This review covers the most significant achievements that have occurred in recent years, but also revisits early discoveries in the field of nano-islands, their fabrication, and optical properties, and later their application as plasmonic sensing platforms. Historically, nano-islands as entities emerged from thin metal film studies. They were generated by the annealing of thin films deposited by physical methods and their formation, driven mostly by the minimization of surface energy. As-deposited thin and very thin metal films are not stable thermodynamically, and by dewetting, will coalesce into small droplets, called nano-islands [1,2,3,4]. Dewetting of metal thin films, showing the evolution of solid structures, is considered an important structure-directing mechanism. Thermally generated nano-islands are random, their size distribution is quite wide, and their adhesion to glass substrates is not strong enough for sensing purposes. The adhesion has been improved by using various under- or overlayers, or by partially embedding them in the glass substrate. Detailed investigations have demonstrated the high sensitivity of nano-islands’ plasmon bands to changes in the surrounding environment, thus making them suitable to sensing purposes. Historically, they were the first to be investigated as plasmonic sensors for localized surface plasmon resonance (LSPR) and surface-enhanced Raman spectroscopy (SERS) applications. Furthermore, many attempts have been made to improve the size and size distribution of nano-islands, especially for LSPR sensing—for example, multiple dewetting, alternating with annealing, or the fabrication of micro-patterned arrays of Au and Ag nano-islands through template confined deposition.

Only in the last decade, after the complete development of synthesis methods of nanoparticles, nano-islands started to be prepared from nanoparticle films using wet methods. There are only a few publications on the fabrication and characterization of these nano-islands; however, they have been used successfully for analytical purposes. Extremely important, under the present circumstances, is the application of plasmonic photothermal biosensors, based on gold nano-islands, for the fast and accurate detection of SARS-CoV-2 [5].

This review paper is structured as follows: Section 1 is dedicated to nano-islands fabricated from thin films and plasmonic sensing based on thermally generated nano-islands. Section 2 describes the nano-island platforms from nanoparticle films, together with their more recent LSPR sensing applications. This section also discusses the work of the authors’ group in the field of plasmonic detection of biomolecules and biological entities, such as bovine growth hormone and exosomes, respectively, and the integration of the sensing methods in a microfluidic environment. We also briefly discuss the most relevant examples of the use of the plasmonic biosensors for real bioanalytical and clinical applications. In this context, the applications of plasmonic biosensors on a chip for point-of-care (POC) are discussed in a separate section.

At the end of the paper, our knowledge on nano-islands and their applications are summarized, and future work in the field that may bring new ideas in the field is emphasized.

## 2. Solid-State Dewetting of Thin Metal Films: Principle and Driving Force

### 2.1. Nano-Island Platforms from Thin Metal Films

Most of thin metal films deposited by physical methods, such as thermal evaporation, sputtering, etc., are not stable structures because they are formed far from equilibrium [1,2,3]. When applying an external energy—for example, heating at temperatures below the melting point of the metal—the thin metal film will transform spontaneously into nano-islands. Other methods to initiate this transformation are ion bombardment, mechanical stress, and laser irradiation (CW (Continuous Wave) or pulsed). The process is called solid-state dewetting or agglomeration, and is driven by the minimization of surface energy, as well as of the free energy associated with interfaces. 

**Figure 1:**
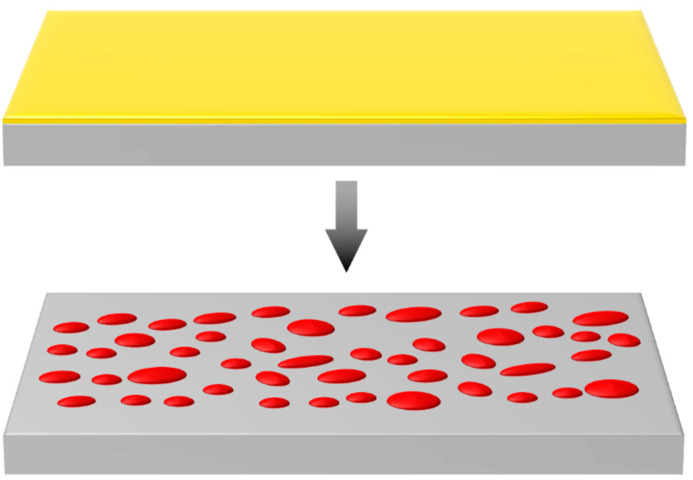
Dewetting of a thin gold film with the formation of nano-islands.

Abbott et al. [4] defined solid-state dewetting as the process by which thin solid films will coalesce into small particles as seen in Figure 1, at temperatures far below the melting point, driven by surface energy minimization. Dewetting of thin metal films showing the evolution of solid structures is considered an important structure-directing mechanism. Work on this topic started to be published in the 1970s, and since then, many studies on the phenomenology of dewetting have contributed to the understanding of its mechanism and pointed to ways toward various applications.

A fundamental study on the thermodynamics and kinetics of film agglomeration was published by Srolovitz and Goldiner in 1995 [6]. They defined agglomeration as the process by which an initially flat continuous film “dewets”, or uncovers a substrate. They analyzed in detail the forces that could contribute to the destabilization of a continuous film, such as surface tension principally but also stresses (especially tensile) within a film. The authors demonstrated that agglomeration (dewetting) is largely a hole nucleation and growth phenomena. They thought that the main mechanism is the heterogeneous nucleation of holes at defects, such as grain boundaries, or even holes left though deposition and processing of the film. As the hole grows, it pushes material out of its way, piling it up along the periphery of the hole.

The process may begin at edges or at preexisting holes in as-deposited films, exposing the substrate. If holes or other defects, such as, for example, contaminants or capping layers in the case of nanoparticle films, do not already exist, they can be formed by ruptures in the film, and the holes will grow when the temperature increases and the growing holes will impinge upon each other.

Three different mechanisms have been proposed to explain dewetting: heterogeneous, initiated from a defect at the film surface; homogeneous, due to a small thermal density fluctuation; and spinoid dewetting, which is typical of liquid films [2,7]. Dewetting leads to the formation, not only of separate objects like droplets, but also stripes and pillars. Depending on the temperature, the substrate of the metal film (especially in the case of very thin films), and the time of annealing, dewetting may be incomplete, in which case no islands are formed. However, the structures formed in this way may be useful—for example, as electrodes in microstructures [8]. 

The principal parameters that affect dewetting of thin metal films are the thickness of the film, the temperature of annealing, and the substrate [9]. It has been recently demonstrated that the temperatures at which nano-islands form vary for different substrate materials [10]. The grain size, in the case of polycrystalline films, may also affect the dewetting phenomenon. The diameter of the nano-islands formed by dewetting depends on the thickness of the film as seen in Equation (1). The following relationship was proposed by Trice et al. [11]:

(1) $$D={[\frac{24\pi 3\gamma }{Af\left(\theta \right)}]}^{\frac{1}{3}}\;h^{\frac{5}{3}}=Ch^{\frac{5}{3}}$$

where *f*(θ) is the geometric factor, based on the particle contact angle θ; γ is the surface tension of the metal; and *A* is the Hamaker constant. This relation was observed in several experimental studies and is valid only at the temperature at which the nano-islands appear, as seen in SEM (Scanning Electron Microscopy) images.

The tendency to dewet depends largely on the surface diffusivity of the metal [3]. Gold has a very high surface diffusivity; therefore, thin gold films will dewet easily, resulting in the formation of nano-islands. However, for some applications, this high tendency to dewet may be a drawback that can be reduced by alloying with metals like Pt or Ti [12].

Both continuous gold films and gold films of thicknesses below the percolated threshold (typically 5–10 nm nominal (mass) thickness) have been dewetted [13]. It has to be mentioned that in the literature there is no general consensus regarding the definition of “thin”, “very thin”, and “ultrathin” films. For the sake of further discussion, films having 1–3 nm thickness will be considered very thin with a well-defined plasmon band, while 4–10 nm will define thin (semi-continuous) films.

**Figure 2:**
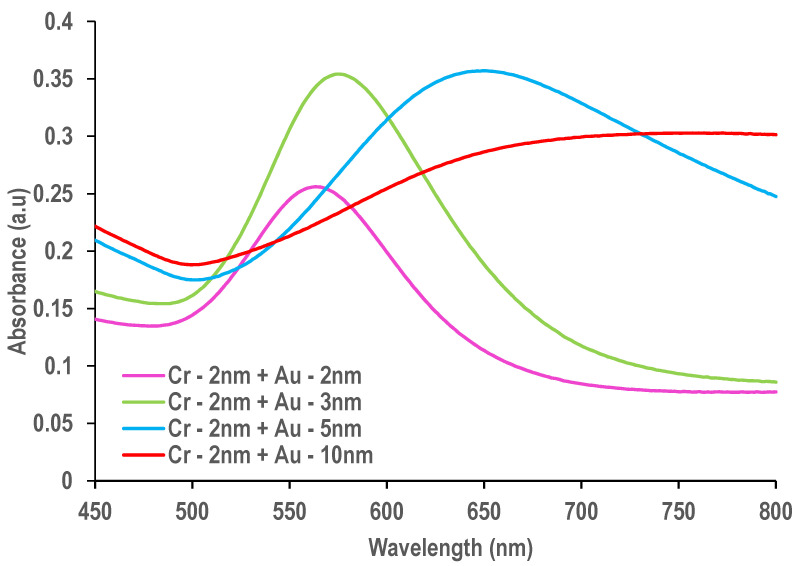
Visible spectra of e-beam evaporated gold films, annealed at 500 °C for 2 h with different nominative film thicknesses of gold (Au) with a 2 nm chromium (Cr) underlayer.

It can be seen in Figure 2 that very thin films (2 nm and 3 nm) show a well-defined plasmon band around 567 nm and 578 nm respectively, corresponding to discrete nanostructures, while the 5 and 10 nm films that are (semi-) continuous show a large plateau toward the NIR (Near Infrared) region of the spectrum.

The authors investigated the transformation from percolated film to islands by in situ transmission spectroscopy, using a temperature-controlled tube furnace and doing measurements all along the annealing process.

Nano-islands have been prepared not only from Au films, but from Ag, Pt, Pd, and Cu as well [14,15]. Large area nano-islands with nanogap spacing have been prepared by multiple dewettings. It has to be mentioned that dewetting the same metal film on different substrates will result in nano-islands with different sizes, showing the important role of the interfacial energy [16].

For example, nano-islands deposited on single-layer graphene by physical vapor deposition or in situ, solution-phase chemical synthesis exhibit completely novel properties. For this reason, it has been called a composite film, which can be regarded a metamaterial [17]. The optical and electronic properties of this material, controlled by the morphology of nano-island films, are reminiscent of semiconductors. The authors emphasized that the role of graphene in this material is not only of support, but principally, graphene is the active component that determines the optical and electronic properties of the composite. The surface energy of the substrate supports that graphene influenced the morphology of the nano-island films, and thus, a wide range of morphologies could be produced, from isolated islands to percolated networks. These graphene nano-island composite films exhibit tunable morphologies and small gaps between adjacent nano-islands, and could be transferred to flexible substrates [18].

The most important applications of metal nano-islands on graphene, especially in sensing, will be discussed in Section 2.1.1. The nano-island graphene composite films contain gaps between the nano-islands, which, when functionalized with benzene thiolate, behave as hot spots for surface-enhanced Raman scattering (SERS). The authors have shown that the mechanical strain in the film increases the sizes of the gaps; this attenuates the electric field, and thus, the SERS signal. This “piezo-plasmonic” effect can be quantified, and the sensor is among the most sensitive mechanical sensors of any type. 

A simple sputtering, annealing, re-sputtering, and re-annealing process was proposed to tune the structural and optical characteristics of Au nano-islands on a glass substrate [19]. 

It was found that the size and inter-particle distance of nano-islands depend on the annealing time and temperature. High temperature annealing tends to increase the size and inter-islands distance of Au islands. Re-sputtering and reannealing under different conditions made possible again the tuning of the size and inter-particle distance. Investigations of the optical characteristics of Au nano-islands demonstrated that the surface plasmon resonance peak of islands was tunable from 510 nm to 620 nm. The authors found that the best results in terms of refractive index sensitivity were obtained when the re-annealing temperature was 500 °C for 5 h. These results suggest that the scheme “sputtering, annealing, re-sputtering, and re-annealing” is an effective method to tune the structure and increase the refractive index sensitivity of sputtered Au islands. 

Different kind of adhesion layers have been investigated to increase the adhesive strength between the gold film and the glass surfaces, without damping the LSPR in gold nano-islands [20,21]. Improvement of the adhesion is commonly achieved by evaporating 5–10 nm Cr, Ni, or Ti underlayers. The metal underlayer is light absorbing, and thus interferes with the absorption signal. Pre-coating the glass substrate with a self-assembled layer of mercapto- or amino-functionalized silane has been shown to improve the metal adhesion. To overcome the disrupting effect of the adhesion layer, dielectric materials are of interest, due to their low absorption of electromagnetic energy. MPTMS ((3-mercaptopropyl) trimethoxysilane) is an alkoxysilane molecule with a thiol functional group. It can be grafted to a glass silicate substrate through a reaction, establishing strong chemical bonds with gold nanoparticles. [22].

A mild annealing (200 °C, 20 h) has allowed the optimization of the optical response [20,21]. Post-coating with an ultrathin, sol-gel-derived silica film was also proposed by Rubinstein’s group as an alternative, to stabilize gold nanostructures formed on silanized glass [23,24].

The authors investigated the optical properties of Au island films prepared by direct thermal evaporation onto indium tin oxide (ITO) substrates, without the need for pre- or post-coating stabilization of the surface. The effect of mild thermal annealing (150 °C, 12 h) or short, high-temperature annealing (500 °C, 1 min) on the morphology of the gold nanostructures was investigated [24]. Because of the poor stability of gold and silver islands in water and various solvents, adhesion layers of Cr and sometimes Ni and Ti are used, especially in sensing experiments [3,24]. The drawback of these materials is their potential diffusion into the Au film during the annealing process [25]. In addition, their possible alloying with gold may result in damping of optical properties [3,26].

Au/Ag alloyed nano-islands with the desired composition have been prepared by thermal dewetting of thin metal films that are thermally evaporated. The complete miscibility of alloyed nano-islands results in the possibility of tuning the plasmon resonance wavelength in the visible range, opening up a new direction for plasmonic enhancement in surface-enhanced Raman scattering or plasmon-enhanced fluorescence. The performance of bimetallic nanostructures may even surpass individual properties and display new properties, often explained by synergistic effects. 

Alloyed nano-islands with well-defined sizes and shapes can be obtained by using successive thermal evaporation of thin Au and Ag films, as well as low-temperature thermal dewetting [27] Successive thin film evaporation and thermal dewetting enable the large-area nanofabrication of Au/Ag alloyed nano-islands with well-defined sizes and shapes. The complete miscibility of Au and Ag leads to tailoring of plasmon resonance over the wavelength region by simply changing the film thickness ratio.

**Figure 3:**
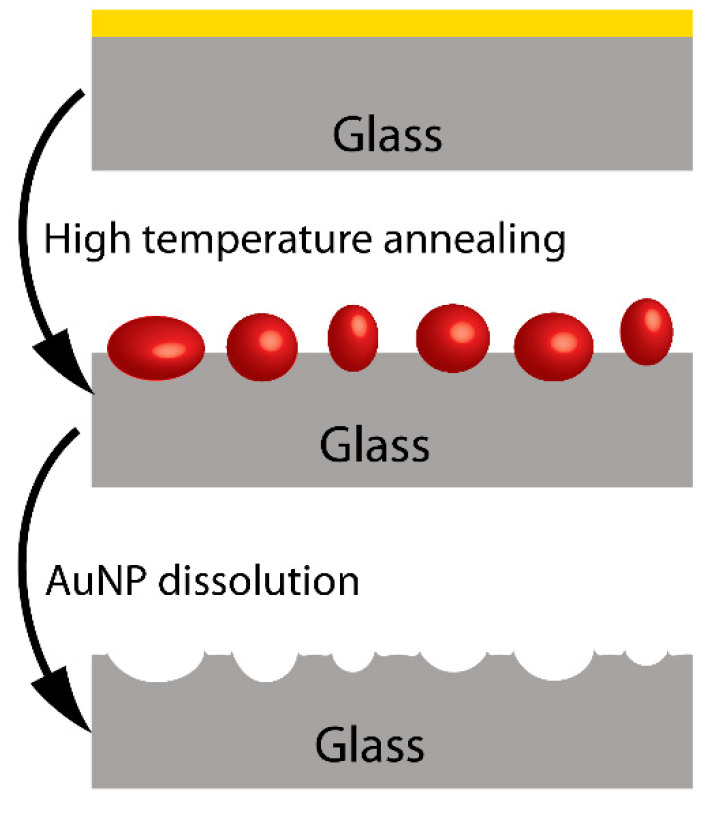
Schematic showing the traces of nano-islands in glass, after their removal by dissolution.

Another method for stabilizing the nano-islands was proposed by Rubinstein group [28], and involves high temperature annealing that results in a partial embedding of the nano-islands in the glass substrate, as shown in Figure 3.

After dissolving the gold, the authors could clearly see the traces of the nano-islands in the AFM (Atomic Force Microscopy) and SEM images. If the nano-islands are not stabilized, immersion in the solvents and drying during the sensing process could result in changes in their morphology, and implicitly, in theor optical properties. Previous authors have explained the poor adhesion of gold and silver to glass and other oxide substrates by their weak chemical interaction with substrates as glass. In order to obtain better adhering nano-islands, dielectric layers of mercapto silane have been used as well [20,29]. 

Other adhering layers, such as the highly transparent and conductive ITO (indium tin oxide) [24] and FTO (fluorine-doped tin oxide) [30] have also been suggested. Ghorbanpour and Falamaki [31] have replaced the Cr and Ti intermediate layers with silver, followed by an annealing treatment.

Post-coating with a very thin silica film was also used to stabilize the gold structure [23]. In another work [24], the authors prepared Au island films by thermal evaporation onto ITO and studied the effect of mild annealing (150 °C, 12 h) or short, high-temperature annealing (500 °C, 1 min) on the morphology of Au nanostructures. They investigated the mechanical, chemical, and optical stability of interfaces. In order to stabilize the islands, SiO_x_ overlayers were also deposited on them. The results have shown that 4 and 6 nm films formed connected the gold structures by dewetting, while 2 nm films formed almost spherical Au nanostructures and small nano-islands. The highest sensitivity was found for the 2 nm film. The authors demonstrated the possibility of real-time sensing by monitoring the absorption of biotin–BSA (Bovine Serum Albumine) and the interaction with avidin.

**Figure 4:**
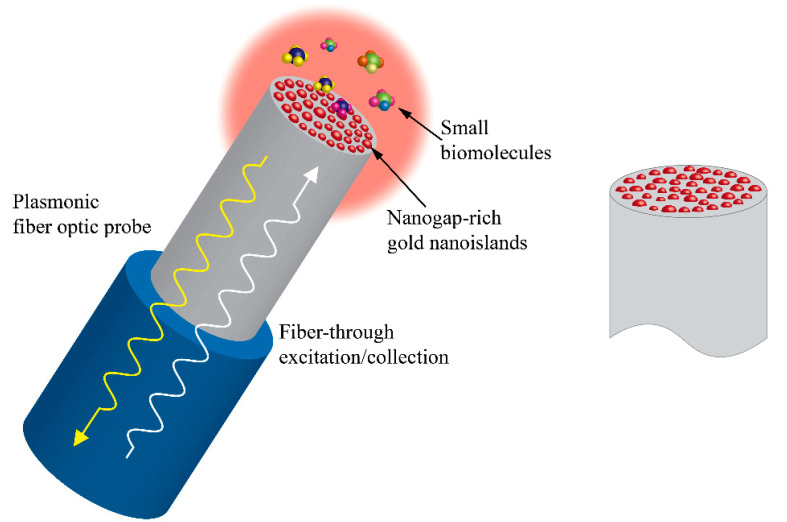
Fiber-optic plasmonic probe for surface-enhanced Raman scattering (SERS) with nanogap-rich Au nano-islands on the top surface of the fiber (modified from Kwak [33], free to read and use under the Creative Commons License).

An interesting technique has been developed by De Almeida et al. [32] and others [33] to grow thin metallic layers on the top of optical fiber surfaces and dewet them thermally to form nano-islands. These configurations, as seen in Figure 4, were used for LSPR and SERS applications and will be discussed more in detail in the following sections of this paper.

#### 2.1.1. Plasmonic Sensing Based on Thermally Generated Nano-Islands

**Figure 5:**
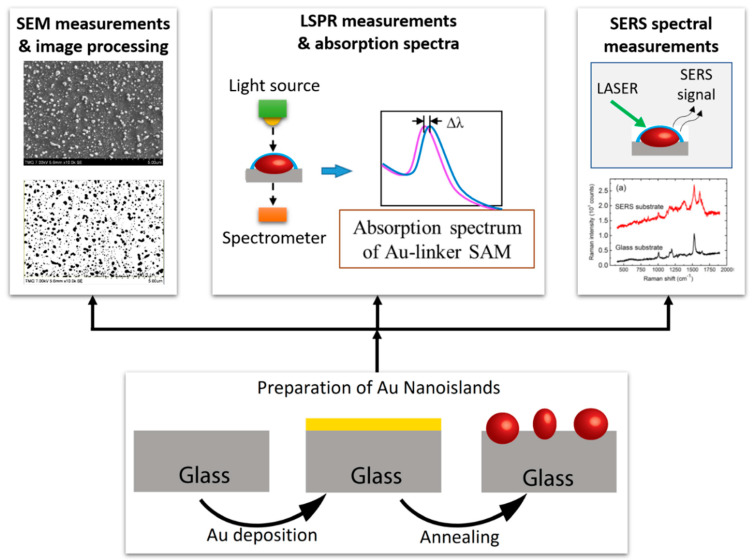
Localized surface plasmon resonance (LSPR) and SERS sensing applications of nano-islands.

The different sensing applications of nano-islands are illustrated schematically in Figure 5.

As shown in Figure 2, very thin films of gold with discrete nanostructures display a well-defined plasmon band in the UV-visible spectrum. After annealing, the random nano-islands formed by dewetting will show a much better defined, narrow LSPR band, situated around 520–530 nm or even at higher wavelengths. It has been found that this Au plasmon band is highly sensitive to the medium surrounding the nano-islands. The plasmon band shifts toward longer wavelengths when the refractive index of the environment is increasing. 

The following equation describes the dependency of the spectral response of the nano-island sensor on the refractive index sensitivity [34]:

*R* = *m*Δη·[1 − exp (−*d/l*)] (2)

where *R* is the sensor response (shift of the plasmon band or/and change in the absorbance), *m* is the refractive index sensitivity, Δη is the change in the refractive index of the surrounding medium due to the binding of the adsorbate, *d* is the thickness of the adsorbate layer, and *l* is the plasmon’s decay length. This rather empirical relationship shown in the Equation (2) can be useful for sensing applications, in spite of the fact that it does not distinguish between absorption and scattering [34].

The sensing performance of plasmonic structures can be characterized by the figure of merit (FoM) = *S_bulk_*/Δλ, where Δλ is the resonance width and *S_bulk_* is the bulk sensitivity, defined as *S_bulk_* = ∂λ/∂*n*—that is, the shift of the resonance wavelength upon a change of the refractive index of the surrounding medium *n*.

The application of thermally-generated nano-islands to sensing was pioneered by Rubinstein’s group [21,34,35,36,37], and subsequently, many other groups have investigated the optical properties and the sensitivity of various platforms for sensing applications [38,39,40,41].

### 2.2. Nano-Island Platforms from Nanoparticle Films

Compared to the studies on dewetting of metal films, there are only a few publications on the fabrication of nano-islands by dewetting nanoparticle films. One explanation that comes to mind is that, historically, dewetting studies were done mostly by physicists, more familiar with physical methods of deposition than with wet methods of synthesis of nanoparticles. They were interested principally in the optical properties of thin films, while chemists’ interest is oriented more toward the fabrication of nanostructures with high refractive index sensitivity for plasmonic sensing applications.

**Figure 6:**
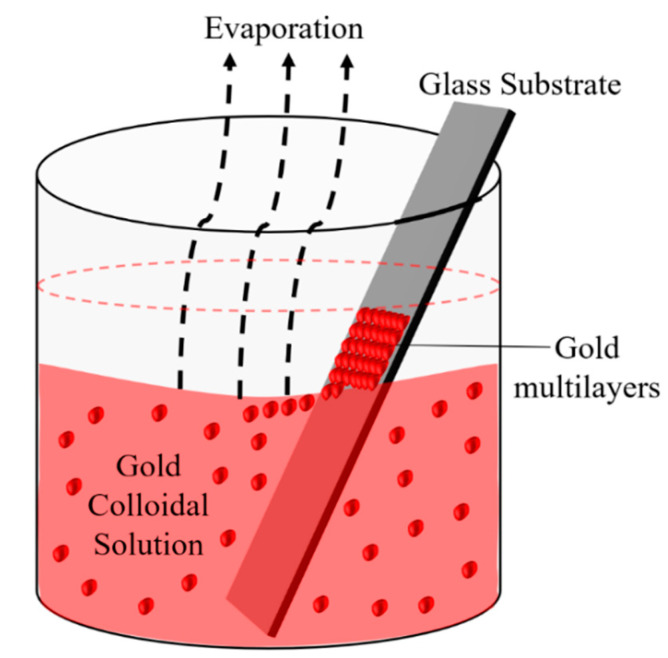
Schematic of the deposition of AuNP (Nanoparticle) multilayers by a thermal convection method (modified from Bathini [42].

Our group principally investigated the analytical performance of nano-islands, prepared by the dewetting of gold nanoparticle films [41,42,43,44,45,46]. The principal motivation for this work was the need for a simple and rapid method of detection of growth hormone levels in milk and milk products. A simple method for the preparation of multilayers of gold nanoparticles on glass substrates was developed by our group by modifying Prevo et al.’s method [47]. The nanostructure obtained from the angled deposition, as shown in Figure 6, has chain-shaped structures with a broad UV-visible absorbance spectrum.

**Figure 7:**
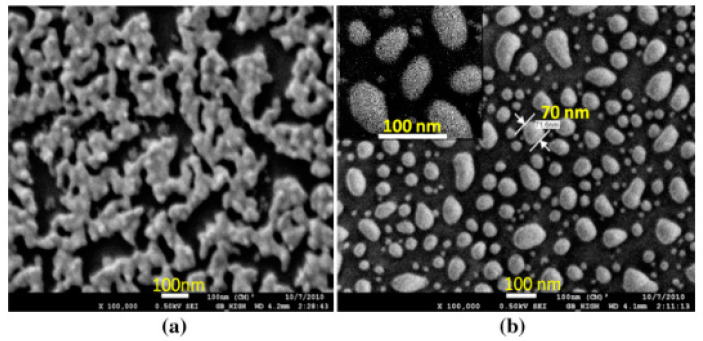
SEM images of three-dimensional (3-D) gold nanostructures (**a**) as deposited and (**b**) after annealing at 550 °C for 1 h (reproduced from [43], open access paper).

The morphology of the non-annealed structures was modified by dewetting to an island-like structure (Figure 7), by annealing at various temperatures. The sensitivity of both non-annealed and annealed platforms was investigated by using solvents with known refractive indices. The sensing results showed a higher sensitivity for the annealed samples—that is, for the nano-island structure. The annealed platform was used for the sensing of bovine somatotropin (bST), using an immunoassay format. The proposed sensing platform showed a detection limit as low as 5 ng/mL of bST. Further, the sensing platform was integrated into a microfluidic device and sensing experiments were carried out. The results demonstrated the suitability of nano-island structures integrated into a lab-on-a-chip device to detect bovine somatotropin with a good sensitivity.

Figure 7a clearly shows the presence of linear aggregates of gold in the sample deposited by thermal convection. After high-temperature annealing, random nano-islands are formed with quite a wide size distribution. The mechanism of dewetting of nanoparticle films is thought to be similar to that of thin metal films, but in this case, dewetting is initiated by defects that originate from capping layers or contaminants. As in the case of metal films, the driving force is the minimization of the energy. 

**Figure 8:**
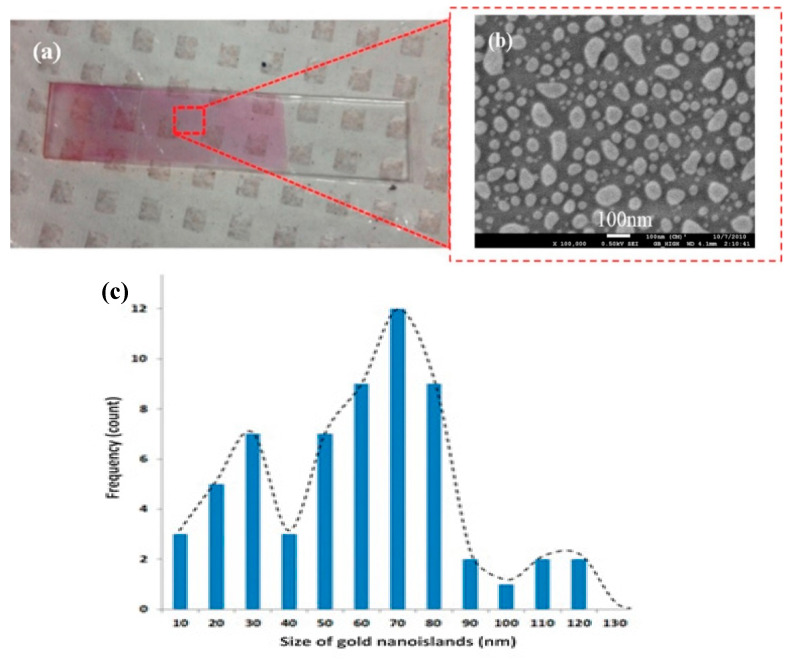
(**a**) Glass substrate with gold nano-islands, (**b**) scanning electron micrograph of gold nano-islands, and (**c**) size distribution of nano-islands (reproduced with permission from [44], copyright 2015, American Dairy Science Association).

The lighter and darker alternating strips seen in Figure 8a originate from the mechanism of the deposition of gold nanoparticles in the case of thermal convection. Because of the various interactions between the nanoparticles and NP–glass substrate, capillarity, etc., the deposition is not continuous. The consequence of this strip-like morphology is the necessity of multiple measurements for at least 4–5 different heights in the beam of the incoming light, and of averaging the positions and absorbances of the gold plasmon band at each step of the sensing protocol.

**Figure 9:**
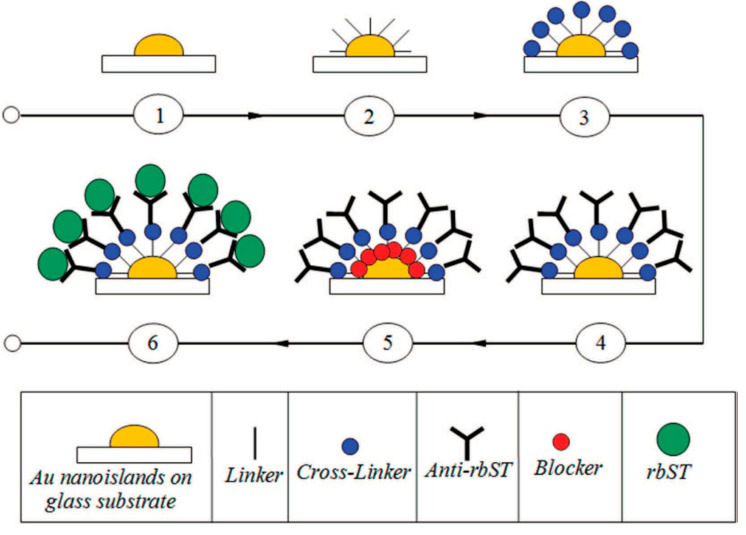
Immunoassay protocol for the detection of recombinant bST (rbST). (1) Sample with gold nano-islands, (2) gold nano-islands with linker, (3) sample after adding cross-linker, (4) sample after adding anti-bST, (5) sample after adding a blocker, and (6) sample after adding rbST (reproduced with permission from [44], copyright 2015, American Dairy Science Association)

The schematic of the immune-sensing protocol for detection of growth hormone is shown in Figure 9. The protocol is similar for the plasmonic detection of other biomolecules and biological entities.

**Figure 10:**
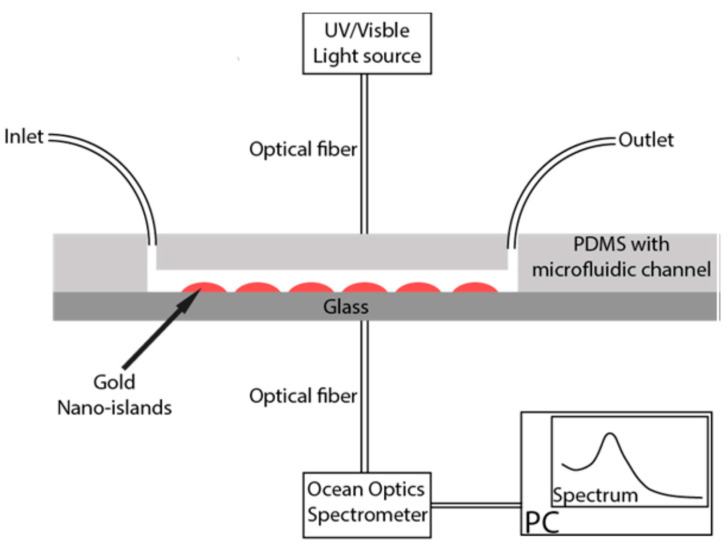
Schematic of the microfluidic device with nano-island structures (reproduced with permission from Int. J. Biomed. Biol. Eng. [50]).

The nano-island structure has proved to be adequate for the detection of bovine growth hormone, by using an immunoassay method based on the localized surface plasmon resonance band of gold. The nano-island structures were integrated into a microfluidic device, as shown in Figure 10, and the spectral measurements were carried out by introducing the device directly in the light beam of an ultraviolet-visible spectrophotometer. A portable assay system using disposable lab-on-chip devices was built as well.

The gold nanoparticles are prepared and deposited on a glass substrate by the thermal convection method, and the gold nano-island structures were formed by annealing of the deposited multilayers of gold nanoparticles [48]. Polydimethylsiloxane (PDMS), the polymer commonly used for microfabrication, is prepared in the ratio of 10:1 by weight with their base and curing agent. PDMS was casted onto a silanized mold to make the microchannel layer. The PDMS layer containing microchannels is bonded with the glass substrate containing the gold nano-islands using oxygen plasma. This microfluidic platform was tested for the isolation and detection of extracellular vesicles (EVs) based on their interaction with Vn96 [49]. Vn96 is a peptide specifically designed to capture EVs by binding to the heat shock proteins present on their surface. 

The fabricated microfluidic device is illuminated with a light source through an optical fiber, and the transmitted light from the device is collected through an Ocean Optics spectrometer connected to a computer. The measured absorption spectrum is analyzed using Spectrasuite software [50]. The schematic of the Ocean Optics spectrometer setup, including the microfluidic device for the detection of EVs based their absorbance measurement, is shown in Figure 10.

A biosensing protocol has been designed and optimized for the isolation of EVs, where various biochemical entities are infused into the fabricated device to functionalize the gold nano-islands to bind Vn96 and capture EVs [50]. Specifically, the first step in any experiment is to measure the absorption spectrum of the gold nano-islands in the collection chamber of the microfluidic channel. Then, a NanoThinks11 solution is infused into the device at a flow rate of 10 µL/min continuously for 10 min, and the spectrum is measured after an incubation of 30 min to observe the shift (Δλ) in the LSPR peak. A positive shift in the spectrum confirms the molecular binding with the gold nano-islands. Then, an EDC + NHS mixture is passed at same flow rate, and the spectrum is measured after incubation. The same procedure is repeated with the streptavidin, biotin–PEG (Polyethylene Glycol) –Vn96. and then EVs. With the adopted protocol, a shift in the peak of the Au LSPR in each spectrum is observed at every stage, confirming the molecular binding of each compound.

Nano-islands were prepared by Feng et al. [51] from a gold colloidal solution mixed with an aqueous solution of polyvinyl alcohol and drop-casted on glass and ITO substrates, respectively. After drying, the samples were annealed at high temperatures for long durations. During the annealing process, the polymer decomposed and all the organic components were removed, leaving on the substrate only the gold nano-islands, without any contaminant.

**Figure 11:**
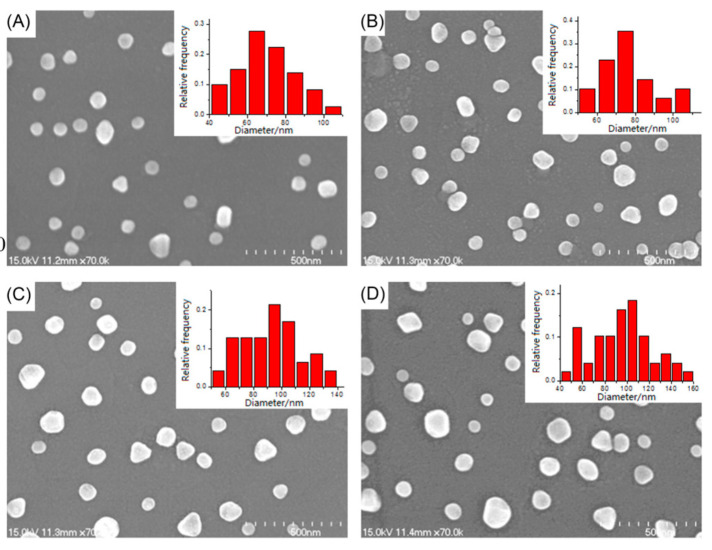
SEM images of AuNPs deposited on indium tin oxide (ITO) glass after annealing at 600 °C for (**A**) 0.5 (**B**) 2.0 (**C**) 4.0 and (**D**) 10.0 h. Inset: size distribution (reproduced from Feng et al. [51], Open Access paper).

As seen in Figure 11, the size of the nano-islands was found to increase with the increasing annealing time, temperature, or volume of PVA (Polyvinyl alcohol) –AuNPs solution. At the same time, the LSPR peak of AuNPs was also gradually red-shifted with the increase in the size of nano-islands, and some hexagonal islands were observed for long annealing times. In addition, the type of substrate surface (ITO or bare glass) also influenced the characteristics of gold nanostructures. The proposed method seems to be a simple and efficient method for the deposition of AuNPs upon bare glass and ITO, as well as fabrication of the corresponding nano-islands.

It was found that higher annealing temperatures (600 °C) and shorter annealing times (0.5 h) lead to higher LSPR refractive index sensitivities, which are strongly connected to the increased island sizes and smaller gap distances. Based on the findings of the plasmonic sensitivity and surface enhancement of thermally generated gold nano-islands, the authors suggested using thicker initial layers and decreasing the inter-particle separation between the particles with shorter annealing times.

### 2.3. Integrated Biosensors for Medical and Clinical Diagnostics

Next-generation healthcare equipment and devices are in great demand for point-of-care (POC), in view of their applications in rapid disease diagnosis, personalized medicine, portable or mobile health care, etc. Localized surface plasmon resonance (LSPR) biosensors, based on noble-metal nanostructures that may provide fast, real-time biochemical detections, have become one of the leading candidates for POC. The advances in nanofabrication, molecular biotechnology, and optoelectronics integration technology have promoted LSPR biosensing to low-cost, easy-to-use, and on-site POC.

The main step in building a device appropriate for clinical diagnosis consists of combining a nanostructure-based LSPR biosensor with a microfluidic device in the most convenient way. As a result, because of the inherent advantages of the microfluidics, the performance of the integrated biosensors is significantly improved. Indeed, a microfluidic device significantly reduces the required sample/reagent volume, and the speed of reactions can be accelerated because of the enhanced diffusive mixing. The chip may have several channels or chambers to process the multiplex detection of analytes of interest, and several separation operations can also be implemented. In addition, miniaturized channel footprints may result in an accelerated antigen–antibody interactions and a fast response.

As shown already, three-dimensional gold nanostructures (nano-islands), fabricated through the convective assembly method, were treated thermally to obtain a nano-island morphology. The nano-island structures were integrated into a microfluidic device, and the spectral measurements were carried out by introducing the device directly into the light beam of an ultraviolet-visible spectrophotometer. A portable device enabling the detection of rbST hormone in milk had been built up. The device can be inserted in a POC device for biosensing, and it could also be used in the future for anti-doping control. A comparison of the sensitivities of the different devices developed in our laboratory shows that the nano-islands-integrated LOC shows the highest sensitivity, that is, the lowest limit of detection. 

However, the POC applications of nano-island platforms can be considered in their infancy, probably because they are subject to less control on the size and distribution of plasmonic enhancement hot spots, chip-to-chip variation, and therefore, low reproducibility for quantitative measurements and continuous monitoring. Many other platforms, such as arrays of nanoparticles with various shapes, nanohole arrays, etc., are today successfully used for POC purposes. The interested reader may see several comprehensive review papers on this topic, including our own papers [52,53,54,55]. Medical applications are rapidly coming to the forefront by combining communications and sensor output to deliver new functions. Devices are able to gather and share information with each other and the cloud. so that data can be collected and analyzed accurately.

In the future, the global system of medical devices will be comprised of a multitude of devices and applications, using sensors, actuators, microcontrollers, and mobile communication devices, and healthcare will be delivered more effectively and at lower cost. It will include not only the collection of patient data for preventive care, but also diagnostics and treatment results. Automation and real-time aspects will reduce errors and improve quality and efficiency. Today, wireless sensor-based systems gather medical data that have never before accessible and deliver care directly to patients. Healthcare will be based on a network of devices that connect directly with one another to capture and share data through a server in the cloud, and then to caregivers. Data captured via sensors will be analyzed, and medical professionals will wirelessly access the information and make treatment recommendations. Remote monitoring means that more patients will have access to adequate healthcare. Progress in sensor technology will, in this way, change the role of hospitals, outpatient sites, and homes. Over the last few years, continuous efforts have been done by researchers in order to develop integrated point-of-care (POC) biosensor networks, capable of automatically delivering results. Biosensors networks are generally a multidisciplinary technological work, based on biological micro electro-mechanical systems (MEMS). The medical needs of the future can be met by using a combination of wireless MEMS and biosensor networks.

## 3. Conclusions and Outlook

The concept of nano-island(s), as it first emerged from early thin film studies, has been described and explained here using the dewetting phenomenon and its mechanism. The literature regarding the fabrication of nano-islands and their optical properties was revisited, with an emphasis on thermally generated islands. Being historically the first, these islands served as models for developing a dewetting mechanism of thin and very thin films under various conditions. Once their enhanced refractive index sensitivity was discovered, it was logical to envisage nano-islands as “building blocks” of the future plasmonic platforms. The first SPR and LSPR sensing platforms were created based on thermally generated nano-islands. Only after the development of the methodology of nanoparticle synthesis did nanoparticle films became the starting point for designing a novel category of nano-islands. In spite of their lack of uniformity in terms of size (diameter and height), these platforms proved, nevertheless, to be highly sensitive, and thus suitable for sensing and biosensing applications. Advanced methods, based on nano-islands integrated into a microfluidic environment, have been described as well. 

Among the emerging plasmonic platforms are the composite materials, based on metal nano-islands on graphene that have extremely attractive electrical, optical, and mechanical properties and a wide range of applications. Novel materials, prepared by transferring the nano-islands to flexible substrates, such as PDMS, are becoming important, especially for point-of-care medical diagnostics. As a general tendency, the morphology and properties of nano-islands on a variety of substrates are now studied for nanophotonic applications. 

Some of the novel applications of nano-islands that enter the current research landscape are oriented toward the enhancement of light absorption in solar cells’ photoactive layers by using a nanoparticle spraying method and the generation of nano-islands by heat treatment [56]. Nano-islands are used more and more to improve the catalytic properties of metals like Pt. The irradiation of the high intensity of light on the Pt film causes the surface energy-driven diffusion of Pt atoms and forms an array of nano-islands on CNT (Carbon Nanotubes) through a photothermal dewetting process [57].

An example of the new, reliable approaches for the fabrication of large-area, periodic metallic structures, including nano-island arrays, are the nano-patterning methods by different template lithography-based techniques [58].

Special gold nanostructures on gold metal films that show a highly sensitive refractive index response, due to the high local field enhancement in their inter particle junction, have been recently reported [59]. It is interesting to mention the use of phase-change materials in tailoring efficient and tunable plasmonic devices [60].
